# Information about peer choices shapes human risky decision-making

**DOI:** 10.1038/s41598-018-23455-7

**Published:** 2018-04-12

**Authors:** Livia Tomova, Luiz Pessoa

**Affiliations:** 1 Department of Brain and Cognitive Sciences, Massachusetts Institute of Technology, Cambridge, MA USA; 2 Department of Psychology, University of Maryland, College Park, USA; 3 Maryland Neuroimaging Center, University of Maryland, College Park, USA

## Abstract

Humans frequently make choices that involve risk for health and well-being. At the same time, information about others’ choices is omnipresent due to new forms of social media and information technology. However, while past research has shown that peers can exert a strong influence on such risky choices, understanding how *information about risky decisions of others* affects one’s own risky decisions is still lacking. We therefore developed a behavioral task to measure how information about peer choices affects risky decision-making and call it the social Balloon Analogue Risk Task (sBART). We tested this novel paradigm in a sample of 52 college young adults. Here we show that risky decisions were influenced in the direction of the perceived choices of others – riskier choices of others led to riskier behavior whereas safer choices of others led to less risky behavior. These findings indicate that *information* about peer choices is sufficient to shape one’s own risky behavior.

## Introduction

In our daily life, we are regularly confronted with decisions that can have serious consequences for health and well-being. For example, choosing whether or not to speed on a highway, participate in drinking games, or have sex with a stranger, all can have major impact on an individual’s life. Thus, understanding how risky decisions are made is of crucial importance. Indeed, the study of risky decision making has been of interest to researchers across several disciplines, including economics e.g.^1,2^, psychology^3,4^, medicine/psychiatry e.g.^5,6^, neuroscience e.g.^7,8^, and biology e.g.^9,10^.

An important focus in the risky decision-making literature is the investigation of peer susceptibility^11–23^, and peer influence has been assessed via multiple approaches. First, analysis of longitudinal and network data^11,12,19,23^ has shown that changes in the behavior of one member of a social network can produce similar changes in the behavior of other members^11,12,19,23^. Second, analysis of self-report measures has shown that the influence of others’ choices on risky behavior outweighs any influence due to changes in one’s perceptions of the benefits and costs of the risky behavior^13^. Third, analysis of behavioral measures of peer susceptibility has shown that peers can influence choices by providing explicit suggestions encouraging riskier play^20–22,24,25^ or by their mere presence^15–18,26^. Lastly, learning about others’ choices in hypothetical scenarios was shown to influence own decisions when others were high-status peers^27,28^. Despite these results, experimental evidence on how *actual choices* of others influence one’s own choices is limited.

Previous studies have assessed how learning about choices of others affects one’s own dichotomous risky decisions (i.e., picking the safe vs. the risky option), and found that participant’s choices were nudged in the direction of peers’ choice^29–31^. Similar findings were observed for decisions outside of risky decision making (i.e., when making dichotomous choices between two everyday objects)^32^. However, a fine-grained measure to quantify the magnitude of peer susceptibility in risky decision-making is lacking so far. In addition, it is unclear how peer influence on dichotomous choices between riskier and safer gambles relates to real-life peer susceptibility in risky decision making.

While neurodevelopmental theories argue that the mere presence of peers increases impulsive reward-seeking in adolescents and young adulthood^33,34^, other hypotheses, such as social norms theory^35^, propose that the direction of the effects (i.e., whether risky or risk averse behavior is encouraged) depends on expectations about appropriate behavior endorsed by a group^27,36^. Empirical research shows support for both hypotheses by showing that the mere presence of peers increases risk taking^15–18,26^ but also showing that risk taking can be affected differently depending on whether the peers present themselves as cautious or risky^29,37^. This is also supported by evidence showing that information about peers’ choices increases the subjective value of this choice for the observer^29^. The present study sought to add new insights into the understanding of how choices of others affect risky decision making by employing a novel paradigm providing a trial-by-trial continuous measure of the magnitude of peer influence.

We modified a laboratory-based behavioral measure of risk taking which defines risk in a simple and direct way^38^. By pumping up a balloon, participants earn money with each pump while the chances of the balloon exploding (and thus the participant not earning money) increase with each pump (Balloon Analog Risk Task BART)^38^. In contrast to other gambling paradigms that require evaluation of probabilities or learning of stimulus-outcome associations, the BART provides a simple and ecologically valid paradigm that correlates with real-life risk taking^38–41^. The task was implemented in a sample of 18-25-year-old college students as emerging adulthood^42^ has been characterized by increased participation in risky behavior, such as binge drinking, drug use, and sexual promiscuity^34^. In college students, the sudden freedom from adult supervision and increased availability of alcohol, drugs, and sexual partners contributes to significant increases in these behaviors^43^. To understand peer influences on risky decision-making, we implemented the BART in a group setting in which participants believed they were finding out about the choices of others while making risky choices themselves.

## Results

**Figure 1:**
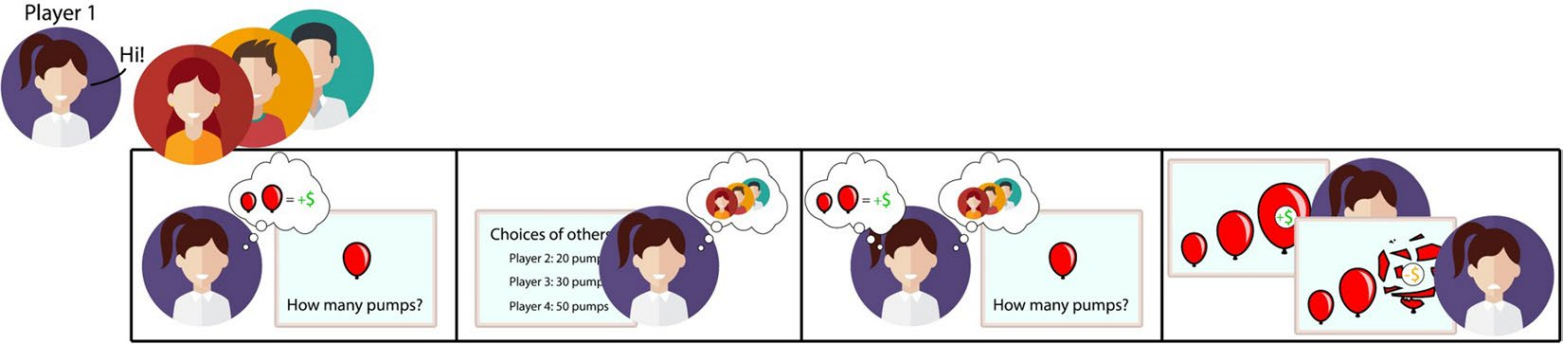
Overview of social BART. Sketch depicting the four different stages in one trial of the sBART. After participants met and read the instructions together, each participant played the task believing that s/he was playing it together with three other participants. On each trial there were four phases: (1) Solo decision: participants decided how many pumps they wanted to inflate the balloon without knowledge about others’ choices; (2) finding about choices of others: participants believed they saw the choices of the other players, and that their own choice was shown to them; (3) informed decision: participants decided how often they wanted to inflate the balloon after learning about the decisions of the other players (they were free to retain the initial number of pumps); (4) outcome: participants viewed an animation of the balloon being pumped up and exploding, or not. Sketch was designed by Christian Meyer using Freepik (http://www.freepik.com/free-vector/people-avatars_761436.htm).

Participants performed the social BART (Fig. 1) where they played two rounds of risky decision making. During the *individual round*, participants were told that the decisions from other players did not matter and could be ignored because we were interested in their individual decision making. During the *team round*, they were told to try to earn as much money as possible for the entire team. As our goal was to determine peer susceptibility to *information* about others’ choices when choices of others were not directly relevant to own choices (i.e., outside of explicit team decision making), we were only interested in the individual round, from which the data were analyzed. Participants performed trials during two risk conditions (high-risk, low-risk) and during filler trials, in which others showed neutral responses. The filler trials were added to increase variance in responses of others and thus the face validity of the task. Each trial was comprised of a *solo decision* (before purported decisions from others were revealed), and an *informed decision* (after purported decisions from others were revealed). The effect of experimental condition (high-risk, low-risk) on the influence index (see next paragraph) was assessed via repeated-measures ANOVA, and *gender* was included as a between-subjects factor to test for possible interactions.

**Figure 2:**
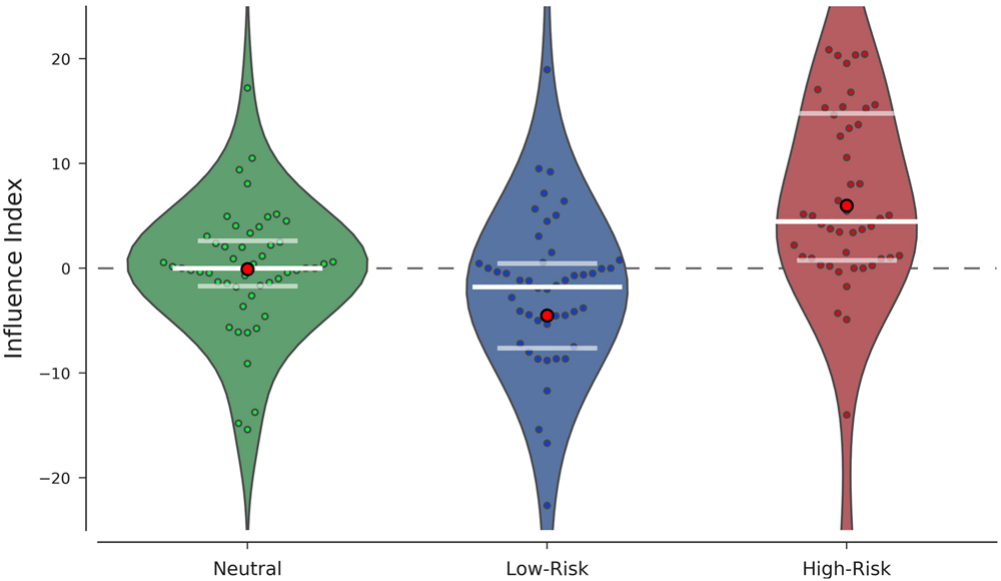
Influence Index. The violin plots illustrate the estimated distribution of the influence index (i.e., change in choice after receiving information on choices of others) in each of the risk conditions (after viewing neutral decisions of others, after viewing low-risk decisions of others, and after viewing high-risk decisions of others). Individual data points in the sample are plotted over the violin plots. The red circles indicate the mean, while the white bars identify the median (bold) as well as the first and third quartiles (faded). The dashed line at y = 0 represents no change between the first and second choice.

The mean number of pumps for solo decisions was 64.04 (SD = 11.86). We calculated an *influence index* by subtracting the pumps chosen on solo decisions from the pumps chosen on informed decisions for each trial and participant. The repeated-measures ANOVA detected a main effect of condition (F(1.430, 71.481) = 16.12, p < 0.001, ƞ_p_^2^ = 0.24, Fig. 2), but no discernible effects of gender (F(1, 50) = 0.757, p = 0.388, ƞ_p_^2^ = 0.02), or interaction between condition and gender (F(1.430, 71.481) = 1.154, p = 0.320, ƞ_p_^2^ = 0.02). Bonferroni-corrected pairwise comparisons (p < 0.017 (0.05/3)) revealed a difference between the high-risk and the low-risk conditions (t(51) = 4.703, p < 0.001), with an increase in the number of pumps for the high-risk condition (mean ± standard error: 5.97 ± 1.92) and a decrease in the number of pumps for the low-risk condition (mean ± standard error: −4.52 ± 1.95). The high-risk condition exhibited a larger number of pumps compared to the neutral condition (mean ± standard error: −0.07 ± 1.36) (t(51) = 4.703, p < 0.001). We also detected a difference between the neutral and low-risk conditions (t(51) = −2.699, p = 0.009).

To further explore potential contributions to decisions, we performed a stepwise regression (forward selection using the Akaike information criterion^44^ for selection; see Methods for details) on trial-by-trial choices. We included the following predictors: (1) participants’ own choice on the previous trial (C_t−1_); (2) participants’ wins on the previous trial (W_t−1_), coded as 1 for win and 0 for loss; condition of the current trial coded via three categorical variables indicating (3) others’ choice was risky on that trial (O_R,t_), or (4) others’ choice was safe (non-risky) on that trial (O_S_,_t_), or (5) others’ choice was neutral on that trial (O_N,t_). We also added the condition (i.e., high risky, low risky, or neutral choices of others) of the previous trial as predictors to assess whether choices of others in the previous trial affected decision making in the current trial (thus including three additional predictors). The best fitting model (see Methods for information on regression steps) for predicting participants’ decisions was a linear combination of low-risk choices of others on the current trial, an interaction of high-risk choices of others in the current trial and own choices on the previous trial, and an interaction of previous outcome and choice on the previos trial (*R*^2^ = 0.10, adjusted *R*^2^ = 0.09, F(7,2999) = 52.9, p < 0.001; Eq. 1):

1 $$choic{e}_{t} \sim 1+{O}_{S,t}+{O}_{R,t}\times {C}_{t-1}+{W}_{t-1}\times {C}_{t-1}$$

We designed our experiment with the goal of contrasting high- vs. low-risk trials. However, others’ decisions (i.e., number of pumps) were viewed as continuous by participants. Thus, we performed an additional trial-by-trial stepwise regression using a continuous predictor of the choices by others on each trial (*O*_*t*_). Specifically, instead of modeling the choices of others categorically (i.e., high risk, low risk and neutral), we calculated the mean number of pumps across others’ choices as this value reflects the information seen by the participant (on each trial, two choices were high-risk, low-risk, or neutral, while one choice was always neutral). As in the analysis of the preceding paragraph, we considered one’s own previous choice (*C*_*t*−1_) and previous outcome (*W*_*t*−1_), in addition to mean choices of others on the previous trial (*O*_*t*−1_). The best fitting model (see Methods for information on regression steps) for predicting participants’ decisions was an interaction of the outcome (win/loss) on the previous trial and one’s own choice on the previous trial (*R*^2^ = 0.06, adjusted *R*^2^ = 0.06, F(4, 3002) = 67.1, p < 0.001; Eq. 2):

2 $$choic{e}_{t} \sim 1+{W}_{t-1}\times {C}_{t-1}$$

Finally, we investigated how behavioral decisions correlated with individual differences in questionnaire-based assessments of real-life risk taking (resulting in 12 scores for each participant). Correlations between behavioral data and questionnaire data were calculated using Spearman correlations. To control for multiple comparisons, we report correlations for p < 0.004 (0.05/12). Solo decisions, which provided an indicator of individuals’ riskiness before learning about choices of others, correlated with Expected Benefits Scale ratings (CARE-R questionnaire) on the scale of risky sexual behavior/new partner (r_s_ = 0.423; p = 0.002). Correlations between informed decisions and individual differences were not detected in our sample.

## Discussion

**Figure 3:**
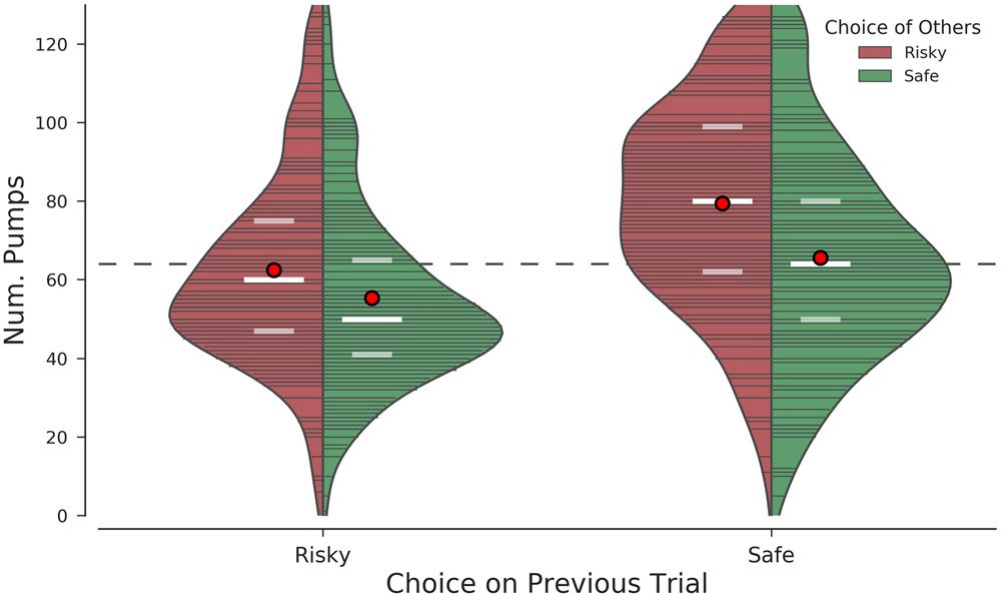
Illustration of the interaction between others’ choice in the current trial and the participant’s previous choice. The violin plots illustrate the estimated distribution of the number of pumps in each condition. Lines running from the center of the split violin to the edge of the density plot indicate individual data points in the sample. The red circles demarcate the mean, while the white bars identify the median (bold) as well as the first and third quartiles (faded). The dashed line at y = 64 represents the ‘baseline’ number of pumps. Participants chose the highest number of pumps on a given trial when their choice on the previous trial was safe and the choices of others were risky on that trial (74 pumps and more coded as risky; 54 pumps and less coded as safe for choice on previous trial).

Here, we sought to investigate how *information about risky decisions of others* influences one’s own risky decisions. To address this question, we developed a novel experimental paradigm designed to investigate peer susceptibility during risky decision making (social BART). We tested our paradigm in a sample of young adult college students as previous literature found increased risk taking in individuals between adolescence and young adulthood^34^^, for review^. Our findings revealed that decisions were influenced in the direction of the perceived choices of others, namely, riskier choices of others led to riskier participant choices, while safe choices of others led to less risky participants. We confirmed this finding in a second analysis on trial-by-trial responses and found that choices were best predicted by a linear combination of low-risk choices of others on the current trial, an interaction of high-risk choices of others in the current trial and own choices on the previous trial and an interaction of previous outcome and choice on the previos trial. In particular, participants’ choice on each trial was dependent on their own previous choice (i.e., how risky or safe they played on the previous trial), on whether they had won or lost on the previous trial, as well as whether others displayed high or low risk choices. As illustrated in Fig. 3, participants chose the highest number of pumps on a given trial when their previous choice had been “safe” and choices of others were “risky” on the current trial.

**Figure 4:**
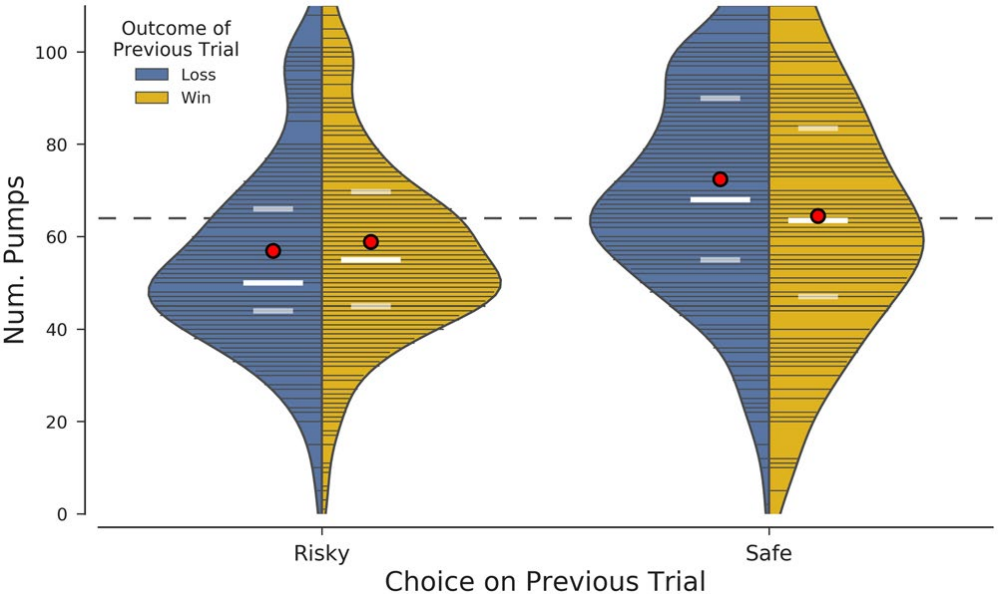
Illustration of the interaction between the outcome of the previous trial (win or loss) and the participant’s previous choice. The violin plots illustrate the estimated distribution of the number of pumps in each condition. Lines running from the center of the split violin to the edge of the density plot indicate individual data points in the sample. The red circles demarcate the mean, while the white bars identify the median (bold) as well as the first and third quartiles (faded). The dashed line at y = 64 represents the ‘baseline’ number of pumps. Participants chose the highest number of pumps on a given trial when their previous choice was safe and had resulted in a loss on that trial (74 pumps and more coded as risky; 54 pumps and less coded as safe for choice on previous trial).

In addition, participants’ choices were influenced by the outcome of their previous trial (win/loss) which interacted with their choice on the previous trial. As illustrated in Fig. 4, participants played riskier on a given trial if their previous safe choice was accompanied with loss (on the previous trial), again indicating strategic decision making. Overall, our results are consistent with the notion that the influence of others’ choices on risky behavior is important^13^. Thus, our findings overall suggest that *information* about peer choices is sufficient to alter one’s own risky behavior.

In a second regression analysis, instead of modeling others’ choices categorically, we used a continuous predictor based on the average number of pumps by others. In this case, the analysis did not indicate that others’ decisions contributed robustly to participants’ choices. Thus, it appears that the magnitude of risky/safe choices of others did not parametrically affect choices of participants. Instead, the direction of perceived choices of others may indicate a preference-congruent susceptibility to social signals of “safety” and “risk,” in line with^29^.

The number of pumps in solo decisions (before decisions from others were revealed) was comparable to the mean number of pumps in a previous version of the BART (M = 60.4)^45^. Thus, despite the social context of our task, participants showed a similar pattern of decision making before putative peer information was provided. Additionally, solo risky choices in the social BART (i.e., decisions made before learning about choices of others) correlated with self-report ratings of risk-taking in real life, indicating that choices in the social BART relate to real-like risky decision making. Interestingly, this association was only found for self-report of risky sexual behavior and not for self-report of heavy drinking or drug use. We speculate that the motivation to engage in risky sexual behavior might be different from that of heavy drinking, or drug use. Indeed, previous research has shown that enhancement motives (i.e., appetitive self-focused motivations, such as having sex to enhance physical or emotional pleasure) were predictors of risky sexual behavior in adolescents and young adults^46^ while heavy drinking was shown to be better predicted by other factors, such as perceived peer drinking norms^47,48^ or negative mood^49^. Furthermore, drug use motivation and alcohol consumption motivation were found to underlie similar motives^50^. However, because these analyses were exploratory, further research is necessary to assess whether the correlation between risky sexual behavior and solo choices in the social BART can be replicated and to illuminate the association between risky decision making in a gambling task and risky sexual behavior in more detail.

Prior versions of the BART reported that females behaved more conservatively than males^38,45^, and previous research found higher self-reported resistance to peer influence in women^51,52^. Here, we did not observe a discernible effect of gender on risky decision-making or on peer susceptibility, which is aligned with previous findings assessing peer susceptibility in risky decision making^29,30^. Previous research has also found that gender differences in risk taking decrease during the transition from childhood to adulthood^53^^, for meta–analysis^. Nevertheless, future research should further assess whether peer susceptibility in risky decision making differs between men and women.

Although we confirmed during debriefing that participants believed that choices of others were not relevant to their own choices during the individual round, a potential limitation of the present study is that the prospect of a subsequent team round may have altered participants’ attention to the choices of others. While we added this round in order to provide an explanation why participants were seeing choices of others, future studies should assess whether our results can be replicated in an experimental design using the social BART without the team round.

In sum, the social BART developed here is well suited to measure peer susceptibility during risky decision-making. The paradigm generates an influence index that allows quantification of the extent to which peer choices affect an individual’s decision making when risk is involved. In addition, it allows the study peer susceptibility on trial-by-trial choices. The task has a simple and engaging setup, a particularly attractive feature for studies with children or adolescents, as well as patient populations. Critically, the task revealed that information about risky choices of others is an important factor in participants’ risky decision making. Understanding how choices of others can influence risky behavior will be increasingly important in light of the ever-growing exposure to information about others’ lifestyles and opinions due to new forms of social media and information technology.

## Methods

### Participants

Fifty-two healthy participants between 18 and 25 years (33 female; M = 20.33, SD = 2.05) were included in the study. The study was approved by the institutional review board of the University of Maryland – College Park, and performed in agreement with the latest revision of the Declaration of Helsinki (2013) regarding the treatment of human research participants. Written informed consent was obtained, and all participants received $10 plus their winnings from the gambling task for participation.

### Procedure

All experimental sessions were performed in groups of four participants. Participants first met and read the instructions for the paradigm together in a group room. Subsequently, participants were accompanied to individual testing rooms and the “social BART” was implemented. Then, participants were given a questionnaire investigating risk-taking in real life^43^. At the end of the experiment, participants were debriefed and received their payment of $10 plus winnings on one randomly chosen trial.

### Social Balloon Analogue Risk Task

We adapted the automatic version^45^ of the BART^38^. All stimuli were presented using Psychtoolbox implemented in Matlab 9.2 (MathWorks) running on a Windows 7 PC. As in the original task, participants were required to pump up a simulated balloon (0–128) pumps. While every pump increased potential earnings of participants, the likelihood of the balloon exploding also increased with each pump. Thus, on every trial, participants’ decisions on how much they pumped the balloon to potentially increase winnings, at the risk of losing everything if they pumped up the balloon too much, provided a measure of risky decision-making. Each balloon exploded at a number of pumps unknown to the participants (explosion points were taken from previous work)^45^. The sequence of explosion points was based on previous work^38,45^, and for each balloon the explosion was equally likely to occur on any given pump, apart from the constraint that within each sequence of ten trials the average explosion point was 64. All participants were presented with the same distribution of explosion points to limit unnecessary variability. Following procedures used previously^45^, and in order to minimize learning across the session, participants were told that choosing 64 pumps represented a typical choice in the task.

We implemented the task in a way that participants believed to be playing with three peers. Thus, the paradigm was implemented as a group experiment and four people were tested simultaneously while believing they were actually seeing the choices of the other participants (which, in reality were predefined choices). Groups of participants were randomly created from participants who signed up for the respective time slots; participants were unknown to one another and included both genders. The interaction with other group members was limited to reading the instructions for the experiment together in a group room. After reading the instructions, all participants were led to individual testing rooms. While they believed they were playing the task together with the rest of the group, participants were presented with computer generated information throughout the experiment. Thus, because participants were not able to influence each other during the experiment, we treated each participant as providing independent data in our statistical analyses. The experiment had two decision-making rounds, an *individual* and a *team* round. The team round provided a cover explanation to participants as to why they would see the choices of others so as to detract from the actual goal of studying peer susceptibility. Thus, participants were told that they would play a gambling task as a team. Furthermore, participants were told that an individual round preceded the team round so that “individual measures” on decision making could be collected. Critically, regarding the individual round, participants were explicitly told that they would see others’ choices because the two types of rounds were designed to match each other in terms of visual input and setup; additionally, they were told that they did not have to pay attention to others’ choices during the individual round, as they would not affect their own earnings in this round.

For the team round, participants were explicitly told that they should try to find a way to earn as much money as possible for the whole team. A fixed order of experimental conditions was implemented: the individual round always preceded the team round. This was done to avoid potential priming effects or other carry-over effects between rounds. For instance, participants might develop a strategy playing as a team that could influence their choices in the individual round. Because we were interested in quantifying peer susceptibility, only the choices in the individual round were analyzed. In other words, strategic decision-making was not the object of study. We confirmed the success of our instructions in a debriefing session at the end of the experiment, as all participants reported believing that choices of others were task irrelevant during the individual round.

On each round, participants performed a series of trials, each of which involved making two decisions. On each trial, they first indicated how much they wanted to pump up the balloon (*solo decision*). After they disclosed their decision, they were presented with the decisions of the other team members (in reality, they were predefined decisions). They were informed that their own decision would be shown to the rest of the team. After seeing the decisions of the team members, participants were asked again how much they want to pump up the balloon (*informed decision*). Participants were told that they could change their initial response or that they could stick with their first response. Following the informed decision, participants viewed an animation of the balloon being pumped up and either exploding or not. In case the balloon did not explode, participants heard a cash register sound, and were presented with visual feedback indicating how many points were won (each point was converted to 32 cents). If the balloon exploded, participants heard a deflating balloon sound together with visual feedback that they did not gain any points. Figure 1 depicts the different stages of each trial.

The experiment included three conditions. In the *high-risk* condition, participants viewed decisions by other players that were high risk (a relatively high number of pumps was chosen). In the *low-risk* condition, participants viewed decisions by other players that were low risk (a relatively low number of pumps was chosen). In the *neutral* condition, participants viewed decisions by other players that were close to the “typical” number of pumps (which they were previously informed to be 64). For each participant, each round included 20 trials per condition (high-risk, low-risk, neutral). Riskiness of decision was categorized as follows: high-risk choices ranged between 75–128 pumps, low-risk choices ranged between 1–53 pumps, and neutral choices ranged between 54–74 pumps. Neutral trials were added to increase variance in the purported choices of others to improve the face validity of our task (i.e., create the appearance that others’ choices came from real participants). Our analyses focused on the difference between high-risk and low-risk trials which were our two key experimental conditions. However, for the sake of completeness, we report results of neutral trials as well. In addition, to increase face validity of our task, on each condition and on each trial, two out of the three choices seen by a participant were high risk, low risk, or neutral; the remaining choice seen was always neutral.

### Questionnaire assessing real-life risk taking

To asses real-life risk taking, we used the revised version of the Cognitive Appraisal of Risky Events questionnaire CARE-R^43^ which consists of three different scales: 1) Past Frequency Scale, investigating the frequency of certain risky behaviors over the course of the previous six months; 2) Expected Benefits Scale, investigating how participants rate the likelihood of positive consequences associated with certain risky behaviors; and 3) Expected Risks Scale, investigating how participants rate the likelihood of negative consequences associated with certain risky behaviors. For those three scales, the questionnaire investigates risk taking in the domain of risky sexual behaviors, drug use, and heavy drinking. Participants rated on a scale of 1 (lowest frequency of 0 times over last 6 months/not at all likely to result in positive consequences/not at all likely to result in negative consequences) to 7 (highest frequency of 31+ times in the last 6 months/extremely likely to result in positive consequences/extremely likely to result in negative consequences). Example questions from the CARE-R are: Please circle the number of times that you engaged in each behavior over the past 6 months: (1) Had sex without protection against sexually transmitted diseases with someone I just met or do not know well; (2) Drank alcohol too quickly; (3) Tried/used drugs other than alcohol.

### Data processing

For each participant, we determined the mean score (i.e., number of pumps) for the solo decisions, which served as a measure of individuals’ risky decision making before learning about others’ choices. Likewise, for each participants and condition (high-risk, low-risk, and neutral), we determined the mean score for the informed decision (i.e., after learning about choices of others). To quantify potential change in choice after being informed about the decisions of others, we calculated the difference between solo decision and informed decision (per condition). The mean value provided an indicator of the influence of others on a participant’s own decision, which we call the *influence index*. Data were preprocessed using Matlab 9.2 (MathWorks).

### Statistical analyses

The effect of experimental condition (high-risk, low-risk, neutral) was assessed via repeated-measures ANOVAs. Two ANOVAs were performed, one using the informed decisions as the dependent variable and one using the influence index as the dependent variable. We included *gender* as a between-subjects factor in the two ANOVAs to test for possible interactions. Greenhouse-Geisser corrections were used when the homogeneity of covariances assumption was violated (as determined by Mauchly tests of sphericity). Data from the risk-taking questionnaire were processed by computing average scores for each scale (Frequency, Expected Benefits, and Expected Risks) for each risk-taking domain: risky sexual behavior (regular partner, risky sexual behavior; new partner, risky sexual behavior), drug use, and heavy drinking, resulting in 12 scores per participant. Correlations between behavioral data and questionnaire data were calculated using Spearman correlations. To control for multiple comparisons, we report correlations for p < 0.004 (0.05/12). Data were analyzed using SPSS v. 20 (IBM) and Matlab 9.2 (MathWorks). The threshold for claiming that an effect was detected was 0.05. Stepwise regression was calculated using the *stepwiselm* function implemented in Matlab. The linear regression model was developed via stepwise forward linear regression. The process started with a constant (the intercept) and at each subsequent step, a predictor was added (from the predefined eight predictors described in the results section) based on the change in the value of the Akaike information criterion (AIC)^44^, while also considering removal of previously selected predictors based on the same criterion. After the intercept, the following steps were produced (adjusted *R*^2^ takes into account the number of predictors in the model):

Inclusion of predictor *C*_*t*−1_ (participants’ own choice on the previous trial; adjusted *R*^2^ = 0.051; Eq. 3):

3 $$choic{e}_{t} \sim 1+{C}_{t-1}$$

Inclusion of predictor *O*_*R*_,_*t*_ (others’ choice was risky on that trial; adjusted R^2^ = 0.083; Eq. 4):

4 $$choic{e}_{t} \sim 1+{C}_{t-1}+{O}_{R,t}$$

Inclusion of predictor *O*_*S*_,_*t*_ (others’ choice was safe, or non-risky, on that trial; adjusted R^2^ = 0.084; Eq. 5):

5 $$choic{e}_{t} \sim 1+{C}_{t-1}+{O}_{R,t}+{O}_{S,t}$$

Inclusion of the interaction between *O*_R*,t*_ and *C*_*t*−1_ and exclusion of these two predictors as independent variables (adjusted *R*^2^ = 0.085; Eq. 6):

6 $$choic{e}_{t} \sim 1+{O}_{S,t}+{O}_{R,t}\times {C}_{t-1}$$

Inclusion of predictor *W*_*t−1*_ (outcome on previous trial, that is, win or loss; adjusted *R*^2^ = 0.086; Eq. 7):

7 $$choic{e}_{t} \sim 1+{O}_{S,t}+{O}_{R,t}\times {C}_{t-{1}}+{W}_{t-{1}}$$

Finally, the inclusion of the interaction between *W*_*t*−1_ and *C*_*t*−1_ and exclusion of these two predictors as independent variables (adjusted *R*^2^ = 0.094; Eq. 8 (same as Eq. 1)):

8 $$choic{e}_{t} \sim 1+{O}_{S,t}+{O}_{R,t}\times {C}_{t-{1}}+{W}_{t-{1}}\times {C}_{t-{1}}$$

The optimal model chosen had the smallest AIC which is essentially a trade-off between model size and model fit^44^. Effect sizes are reported as ƞ_p_^2^ for the ANOVA and adjusted *R*^2^ for the regression analysis.

The analysis based on a continuous predictor of others’ choices employed 6 predictors. After the intercept, the following steps were produced:

Inclusion of predictor *C*_*t*−1_ (participants’ own choice on the previous trial; adjusted *R*^2^ = 0.051; Eq. 9):

9 $$choic{e}_{t} \sim {1}+{C}_{t-{1}}$$

Inclusion of predictor *W*_*t−1*_ (outcome on previous trial, that is, win or loss; adjusted *R*^2^ = 0.052; Eq. 10)

10 $$choic{e}_{t} \sim {1}+{C}_{t-{1}}+{W}_{t-{1}}$$

Inclusion of interaction *W*_*t−1*_ and *C*_*t−1*_ and exclusion of these two predictors as independent variables (adjusted *R*^2^ = 0.062; Eq. 11 (same as Eq. 2)):

11 $$choic{e}_{t} \sim {1}+{C}_{t-{1}}\times {W}_{t-{1}}$$

For completeness, we ran a separate model with just others’ choices. The results were as follows. For a model with only *O*_*t*_ *R*^2^ = 0.026; for a model with only *O*_*t*−1_ the best fitting regression model was a constant (thus, *O*_*t*−1_ did not result in changes in model fit); for a model with the interaction of *Ot* and *O*_*t*−1_ the adjusted *R*^2^ = 0.031.

### Data availability

The datasets generated and analyzed during the current study are available from the corresponding author upon request.

### Code availability

The code used for running the social BART and analyzing the data is available from the corresponding author upon request.
